# Four-Year Emicizumab Treatment in an Elderly Patient With Acquired Hemophilia A: A Case Report on Perioperative Management Along With a Literature Review

**DOI:** 10.7759/cureus.88625

**Published:** 2025-07-23

**Authors:** Tomoko Yamaguchi, Kagehiro Amano, Yushi Chikasawa, Masato Bingo, Ei Kinai

**Affiliations:** 1 Department of Laboratory Medicine, Tokyo Medical University, Tokyo, JPN

**Keywords:** acquired hemophilia, emicizumab, hemostatic control, long-term outcome, surgery of the stomach, thrombotic events

## Abstract

Acquired hemophilia A (AHA) is a life-threatening bleeding disorder caused by autoantibodies against coagulation factor VIII (FVIII). While immunosuppressive therapy (IST) can eradicate these autoantibodies, it may fail or cause adverse events, especially in elderly patients. Clinical trials involving AHA patients have confirmed the efficiency of emicizumab, a bispecific antibody mimicking FVIII widely used in congenital hemophilia A with or without inhibitors, but the long-term safety and effectiveness in perioperative hemostatic management remain unclear. These issues are important due to the high risk of thrombosis in AHA patients. This report details four years of continuous use of emicizumab and perioperative management in an elderly woman in her 90s with IST-resistant/intolerant AHA. She was diagnosed with AHA one year before the initiation of emicizumab. After initially responding to steroid pulse therapy with a tentative fall in FVIII autoantibodies, she experienced recurrences and repetitive episodes of massive cutaneous bleeding within a year during corticosteroid tapering, which required continuous high-dose steroid therapy. Emicizumab was initiated via enrollment in the AGEHA clinical trial. During the four years of continuous use (the longest reported duration), no major bleeding or adverse events were recorded. The patient underwent emergency surgery for appendicitis, during which hemostasis was maintained with recombinant activated factor VII (rFVIIa). Despite minimal intraoperative blood loss, prolonged drain-site bleeding occurred, possibly due to low emicizumab levels indicated by FVIII activity measured by chromogenic assay. rFVIIa was tapered gradually to postoperative day 27 without thrombotic complications. This case highlights the utility of emicizumab in IST-resistant AHA patients by achieving effective hemostasis and safety against IST-related toxicity. It also demonstrates the beneficial effects of rFVIIa in hemostasis during surgery, emphasizing the importance of careful monitoring in balancing bleeding and thrombotic risks.

## Introduction

Acquired hemophilia A (AHA) is a rare and life-threatening bleeding disorder caused by inhibitors against coagulation factor VIII (FVIII). Treatment of AHA involves long-term immunosuppressive therapy (IST) to eliminate FVIII inhibitors. While IST induces complete remission in approximately 70% of AHA patients, around 15% of those responders experience relapse [1,2]. Another aspect of IST is the relatively long time required to achieve complete remission (median: >5 weeks), during which 25-40% of patients develop side effects, including opportunistic infections, organ dysfunction, and other adverse events that are often life-threatening, especially in elderly patients [1,2].

Emicizumab, a recombinant humanized bispecific monoclonal antibody, is used to replace the function of activated FVIII by binding to both activated factor IX and factor X. It is currently widely used for the prevention of bleeding in patients with congenital hemophilia A with or without inhibitors. Emicizumab has been reported to be effective in the management of AHA [3], and clinical trials have been completed in Europe (GTH-AHA-Emi) [4] and are ongoing in the U.S. (AHAEmi, NCT05345197). In Japan, emicizumab was approved for AHA based on the results of the Japanese Phase III clinical trial AGEHA study [5]. The AGEHA study reported that emicizumab does not cause major bleeding regardless of elimination of autoantibodies, suggesting that it has potent hemostatic activity in patients with IST-resistant AHA [6].

However, a few issues regarding the long-term use of emicizumab remain unresolved, including thrombosis risk, perioperative management, and optimal dosing in elderly patients. In the AGEHA study [5], deep venous thrombosis (DVT) was observed in one of 12 (8.3%) patients during the observation period (44.5 (8-639) days). In addition, although emicizumab can potentially cause thrombosis or thrombotic microangiopathy (TMA) when used in combination with certain bypassing agents in congenital hemophiliac patients treated with inhibitors [7], its safety in AHA patients has not yet been confirmed. Furthermore, little is known about the effects of emicizumab on perioperative hemostasis, where the combination of emicizumab and bypassing agents is inevitably needed. Here we describe an AHA patient on long-term emicizumab therapy who received uneventful perioperative management while on adjunct bypassing agent therapy.

## Case presentation

**Figure 1:**
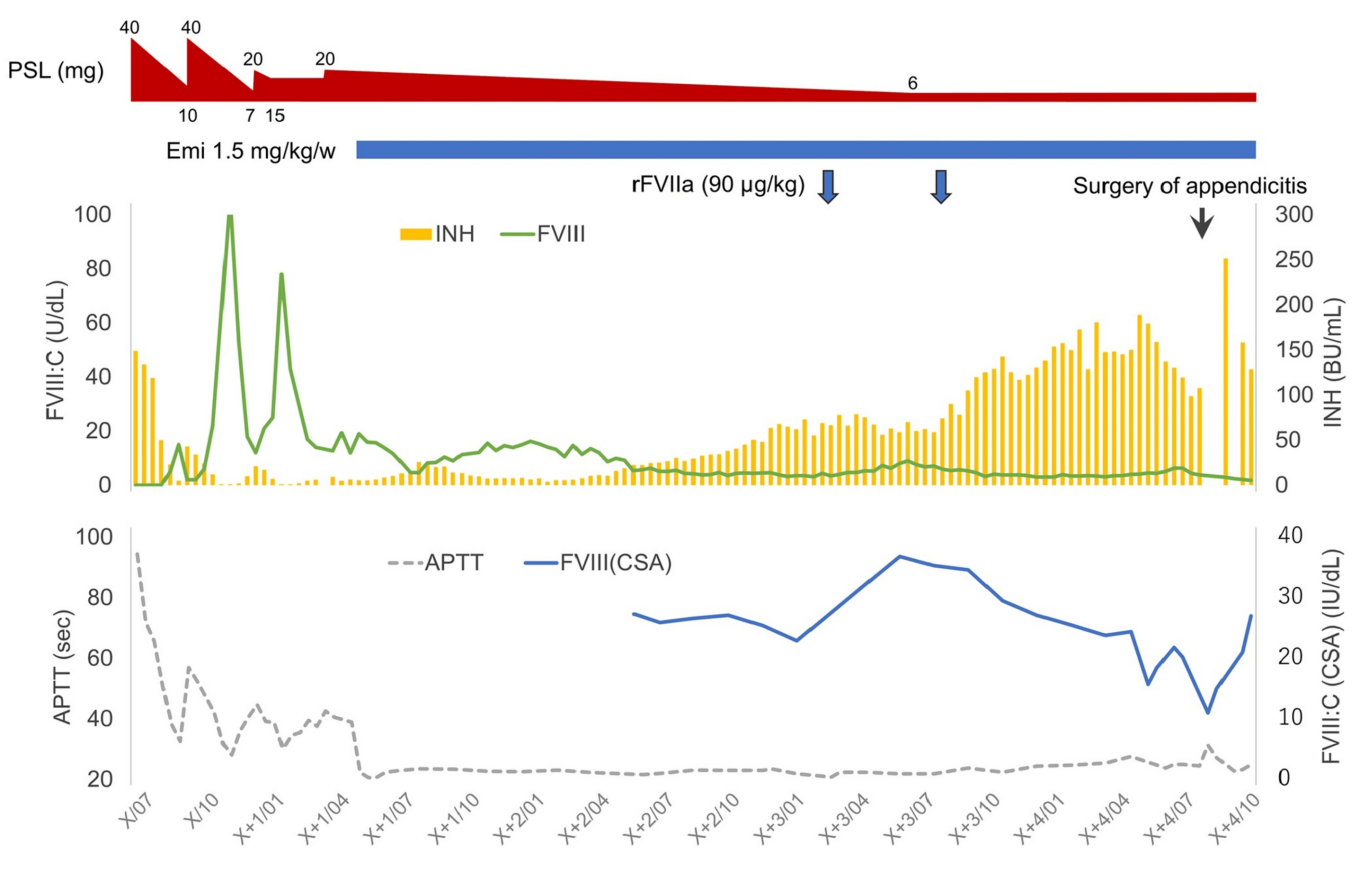
Clinical course. The patient was treated with emicizumab for acquired hemophilia A for over four years. Two single doses of rFVIIa were administered for minor trauma during the course of treatment. FVIII:C: activity of factor VIII, INH: inhibitor of factor VIII, FVIII(CSA): activity of factor VIII measured by chromogenic substrate assay, PSL: prednisolone, Emi: emicizumab, rFVIIa: recombinant activated factor VII, APTT: activated partial thromboplastin time.

The patient was a female in her 90s with a medical history of various autoimmune diseases, including polymyalgia rheumatica, myasthenia gravis, and Hashimoto's disease. One year before the initiation of emicizumab, she was diagnosed with AHA based on the development of a massive subcutaneous hematoma. Immediate initiation of steroid pulse therapy resulted in a tentative fall in FVIII inhibitors from 149 BU to 5 BU over 50 days. However, during the tapering of corticosteroids, she experienced relapses three times in a single year, which required continuation of high-dose steroid therapy (Figure 1). In each relapse, she experienced massive subcutaneous bleeding that progressed to cause anemia, which required treatment with bypassing agents. Moreover, the prolonged use of corticosteroids led to reduced physical activity and increased blood glucose and HbA1c levels. The low activity of daily living (ADL) discouraged the introduction of additional IST.

Due to the poor response and adverse effects of corticosteroids, emicizumab was initiated to prevent bleeding via enrollment in the AGEHA clinical trial. Since the commencement of emicizumab, she did not experience any adverse events over a period, and the occasional minor trauma-related bleeding episodes were well managed by a single dose of rFVIIa (Figure 1). Based on this response, corticosteroid was gradually tapered from 20 to 6 mg (the latter dose was necessary in order to control the pre-existing autoimmune diseases), with the persistence of a high level of inhibitors (58.9 BU/mL) and low level of FVIII (2.3%), as confirmed by emicizumab-neutralized one-stage assay. During the same period, no clinical signs suggestive of thrombotic adverse events were recorded, and D-dimer levels remained within the normal range during follow-up.

**Figure 2:**
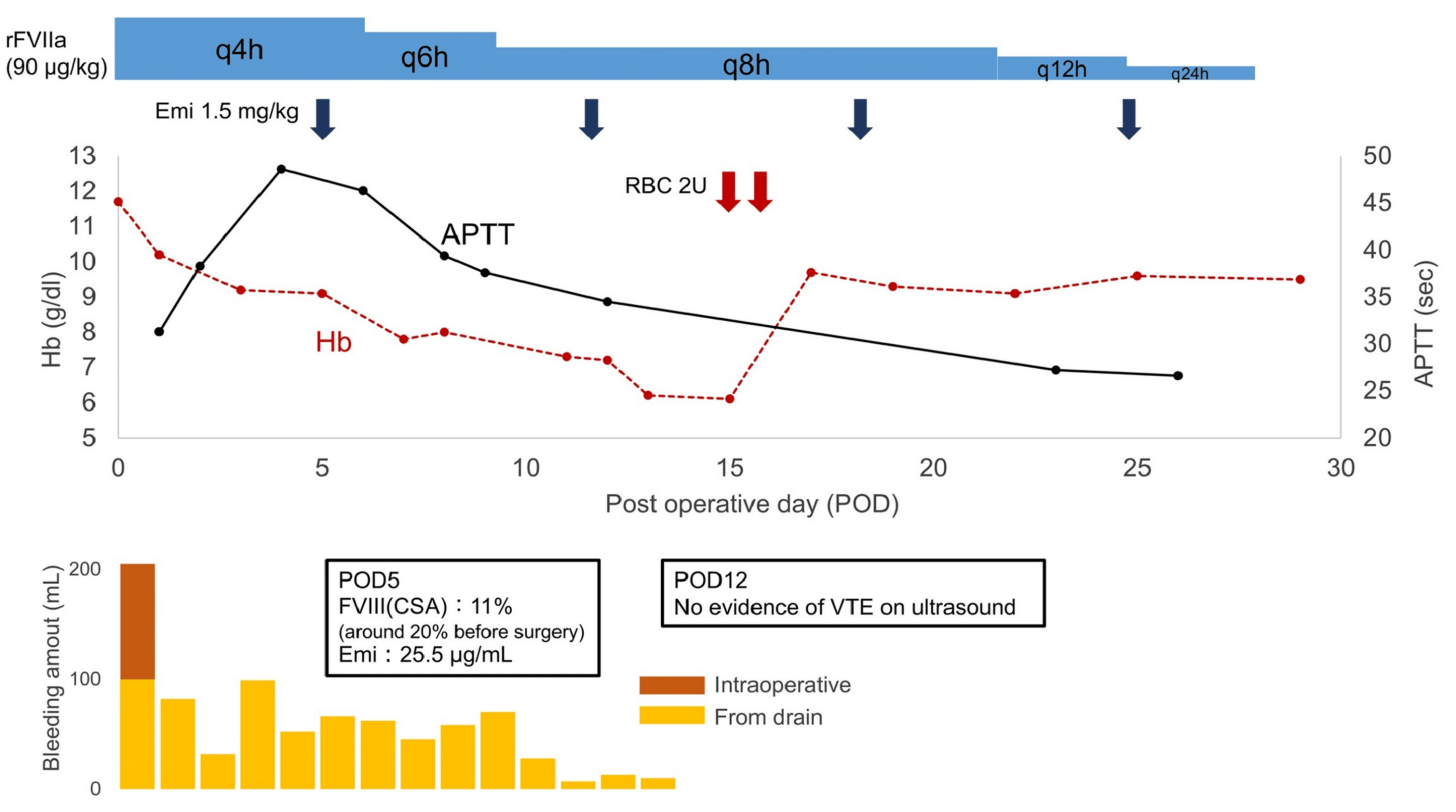
Perioperative management with bypassing agent and hemostatic laboratory values. Marked prolongation of APTT and a decrease in factor VIII activity measured by hCSA at postoperative day four (from 30 to 48 sec, 20 to 11%, respectively), suggestive of a decline in plasma emicizumab concentration due to perioperative blood loss, which caused further prolongation of bleeding and worsening of anemia. POD: postoperative day, rFVIIa: recombinant activated factor VII, Emi: emicizumab, FVIII(CSA): activity of factor VIII measured by human reagent-based chromogenic substrate assay, Hb: hemoglobin, VTE: venous thromboembolism, APTT: activated partial thromboplastin time, hCSA: human reagent chromogenic assay.

Two and a half years after the initiation of emicizumab, she was admitted to the hospital for acute appendicitis with necrosis and peritonitis. An emergency laparoscopic appendicectomy was performed. For hemostasis, 90 µg/kg of rFVIIa was administered before surgery, followed by a repeat dose every four hours (Figure 2). Intraoperative bleeding was 200 mL, which was considered larger than the average volume lost during appendicectomy, typically around 5 to 30 mL [8]. Furthermore, blood leaked persistently through the drainage tube up to 12 postoperative day (POD), which caused anemia and required blood transfusion on 15 POD. Human reagent chromogenic assay (hCSA)-reported FVIII activity and activated partial thromboplastin time (APTT) changed postoperatively relative to the preoperative values (from 20.2 to 11% and from 30 to 48 sec, respectively). The plasma concentration of emicizumab at five POD was 25.5 µg/mL, which was somewhat lower than that reported in other AGEHA emicizumab-treated AHA patients (mean: 37.4±14.3 µg/mL, ±SD) [5]. After regular administration of emicizumab at the same previous dose (1.5 mg/kg) on five POD, hCSA-reported FVIII activity returned to the preoperative level. With regard to the management of persistent anemia, the dose of rFVIIa was tapered gradually: every four hours from zero to six POD, every six hours from seven to nine POD, every eight hours during 10-21 POD, every 12 hours during 22-24 POD, and every 24 hours from 25 to 27 POD. The total number of rFVIIa doses was 99, and the cumulative dose was 8910 µg/kg. No evidence of DVT was noted, as confirmed by ultrasonography on 12 POD, and no other thrombosis or thrombotic microangiopathy (TMA) was encountered during the clinical course.

## Discussion

The present case provides new and valuable information on emicizumab use in patients with AHA. It highlights: (1) the long-term efficacy and safety of emicizumab in patients with IST-refractory and -intolerant AHA, and (2) the importance of careful perioperative hemostatic management of emicizumab-treated patients.

Long-term treatment with emicizumab in patients with congenital hemophilia A (HA) has been shown to be effective in the control of bleeding, with 82.6% of patients achieving zero bleeds and no major drug-related adverse events [9]. However, the long-term efficacy and safety of emicizumab in AHA remains elusive, since the median duration of treatment in the Japanese clinical trial of emicizumab in AHA (AGEHA study) [6] was only 44.5 (8-639) days. Our case report describes the safety of long-term use of emicizumab (over four years) in AHA patients. More importantly, emicizumab allowed reducing the steroid dose in our patient, which improved quality of life and physical activity by avoiding the adverse effects of corticosteroids. Furthermore, the frequency of outpatient visits could be extended to monthly or longer intervals, thereby reducing the overall treatment burden. While the efficacy of emicizumab in IST-intolerant AHA patients was reported in cohort 2 of the AGEHA study, the number of enrolled patients so far is small (n=2), thus requiring further verification [6].

To our knowledge, there is limited information on perioperative hemostatic management of emicizumab-treated AHA patients undergoing major surgery. For emicizumab-treated congenital HA patients with inhibitors, rFVIIa is typically the first-choice bypassing agent, since the use of additional activated prothrombin complex concentrate (aPCC) is often associated with thrombosis and thrombotic microangiopathy (TMA) [7]. Expert opinion on congenital HA recommends rFVIIa at 90 µg/kg every two to three hours in the early postoperative period. However, it is not clear whether this strategy is directly applicable to AHA [10].

AHA patients face a relatively higher risk of thrombosis (reported previously at 4.4%) [2]. Furthermore, thrombotic adverse events have been reported with both rFVIIa (2.9%) and aPCC (4.8%) when used for hemostatic management of AHA even without emicizumab [11]. Given this background, our patient was carefully managed with rFVIIa, employing a wider administration interval (every four hours) compared to the two- to three-hour interval typically recommended for congenital HA with inhibitors [10]. The prolonged postoperative bleeding from the surgical wound drain observed in our patient was probably caused by (1) insufficient amounts of rFVIIa, (2) delayed wound healing due to the prolonged administration of high doses of corticosteroid over several years, and/or (3) low emicizumab blood levels due to the invasive surgical procedures. As discussed below, the observed decrease in hCSA-reported FVIII level suggested a fall in emicizumab plasma levels. Accordingly, we recommend the use of bypassing agents and reloading of emicizumab in major surgery, especially when emicizumab concentration is deemed to decrease by perioperative blood loss.

It is often difficult to measure the coagulation activity of emicizumab in routine clinical practice. Although the thrombin generation assay (TGA) is the most consensus method for the evaluation of Emi coagulability [12], this assay has inherent problems, such as poor result stability, time-consuming, and, above all, it is not available in many laboratories. The clot waveform analysis (CWA) is another alternative assay with useful clinical diagnostic value [13]; however, it exhibits limited efficacy in compensating for inter-reagent and inter-instrument variability.

On the other hand, FVIII activity measured by hCSA correlates strongly with plasma emicizumab concentrations, and such correlation has the advantage of being versatile and yielding stable results. In fact, we reported recently the close correlation of FVIII activity measured by hCSA with emicizumab concentration [14]. While this validation result was based on an analysis of in vitro data, we are currently analyzing the same relationship in vivo. As discussed above, the brief worsening of bleeding observed postoperatively in our patient was assumed to be the result of a decrease in emicizumab concentration, inferred from the decrease in FVIII activity measured by hCSA. However, emicizumab plasma levels may also be affected by changes in fluid distribution resulting from hemorrhage and/or fluid supplementation, or inflammation. In our patient, hCSA-based FVIII activity returned to the baseline value after continuing regular dosing without reloading. Further studies are needed to determine the optimal method of administration.

Reports of surgical AHA patients treated with emicizumab are limited to brief descriptions within clinical trials or case reports (Table 1) [4,6,15-20]. A literature search for articles published between 2019 and 2024 identified wide patient demographics and surgical procedures (both minor and major surgeries). Our case represented a major emergency surgery, similar in invasiveness to cases of pancreaticoduodenectomy (case 7) and small bowel resection (case 9) (Table 1) [4,18]. In these cases, FVIII activity and autoantibodies' levels at the time of surgery were not determined. In case 7 (Table 1) with pancreaticoduodenectomy, rapid postoperative remission of AHA was observed, and good hemostasis was achieved with an only single dose of rFVIIa at 90 µg/kg. Its remission of AHA was likely related to the removal of the underlying malignancy [17]. In case 8 (Table 1), despite the minor nature of the procedure (tooth extraction), the patient experienced significant bleeding, requiring a total of 130 mg of rFVIIa to achieve hemostasis [18]. On the other hand, case 6 (Table 1) developed stroke after a single dose of rFVIIa at 90 µg/kg administered for minor surgery, underscoring the high risk of thrombosis [16]. This heterogeneous response based on the dose of rFVIIa stresses both the challenging task of selecting a standardized regimen and the importance of dose titration to individual patients. The presence of coexisting conditions such as malignancy or autoimmune diseases can further complicate management, potentially increasing both thrombotic and bleeding risks. Our strategy of a wider interval (every four hours) was a deliberate attempt to mitigate thrombotic risk, consistent with the cautious approach required in AHA patients.

**Table 1:** Published surgical cases of AHA patients treated with emicizumab. Cases 7, 9, and 10 (this case) underwent major surgery. Case 6 had thrombosis (stroke), and case 8 had abnormal bleeding. Major surgery involves entry into major body cavities, requires general anesthesia, and carries significant operative time and risk. Minor surgery is limited to superficial tissues, is typically performed under local anesthesia, and involves minimal risk and short duration. M: male, F: female, NM: not mentioned, PCI: percutaneous coronary intervention, VAC: vacuum-assisted closure, rFVIIa: recombinant activated factor VII.

Case	Age	Gender	Type of surgery	Major/minor	FVIII activity (IU/dL)	FVIII inhibitor (BU)	Coagulation factor concentrate	Abnormal intrasurgial bleeding	Thrombosis	Reference
1	87	M	Clipping for colonic bleeding	Minor	<1.0	NM	Y (for bleeds, not for surgery)	No	No	[6]
	87	M	Clipping for colonic bleeding	Minor	11.6	NM	Y (for bleeds, not for surgery)	No	No	[6]
2	77	F	Endoscopic papillotomy	Minor	8.6	NM	N	No	No	[6]
	77	F	Endoscopic biliary dilation	Minor	8.6	NM	N	No	No	[6]
	77	F	Cauterization	Minor	14.9	NM	Y (for bleeds, not for surgery)	No	No	[6]
3	50	F	Cold snare polypectomy	Minor	20.5	NM	N	No	No	[6]
4	72	M	PCI	Minor	<1	409	N	No	No	[14]
5	90	M	Arthrotomy	Minor	NM	NM	N	No	No	[15]
6	79	F	The change of a vacuum-assisted closure (VAC) suction system	Minor	<1	NM	Y (rFVIIa 90 µg/kg)	No	Yes (stroke)	[16]
7	78	M	Pancreaticoduodenectomy	Major	NM	NM	N	No	No	[17]
8	21	F	Tooth extraction	Minor	NM	NM	Y (rFVIIa 130 mg, after abnormal bleeding)	Yes	No	[18]
9	NM	NM	Small bowel resection	Major	NM	NM	NM	NM	No	[4]
10	90	F	Appendicectomy	Major	2.3	58.9	Y (rFVIIa 90 µg/kg q4h and tapering)	No	No	This case

Our study has certain limitations. Our case report describes a single measurement of plasma emicizumab level during the clinical course, performed only on POD five. Due to the patient's clinical condition, measurement of baseline emicizumab levels under stable conditions was not feasible, which limited direct comparison. Furthermore, global coagulation assays, such as TGA and rotational thromboelastometry (ROTEM), were not conducted in this case. Global coagulation assays and plasma emicizumab levels should be incorporated to better correlate clinical bleeding risk with laboratory parameters, and should be performed across a large number of cases. Despite these limitations, our case report provides valuable insights, especially when considered in the context of the limited existing literature.

## Conclusions

In conclusion, our case suggests that emicizumab is useful in AHA patients by achieving efficient hemostasis as well as improving quality of life, and is also safe, with minimal IST-associated toxicity. This case further underscores the critical importance of meticulous perioperative management in surgical patients, given the potential for significant falls in plasma emicizumab concentrations, which may predispose to massive perioperative blood loss. Our case, together with previously reported cases, emphasizes the complexity of management and the need for careful individualized hemostatic management strategies that balance the risks of bleeding and thrombosis in emicizumab-treated AHA patients undergoing surgery. Further multicenter studies and registries are essential to develop standardized perioperative protocols for emicizumab-treated AHA patients.
